# Prognostic value of pretreatment serum alanine aminotransferase/aspartate aminotransferase (ALT/AST) ratio and gamma glutamyltransferase (GGT) in patients with esophageal squamous cell carcinoma

**DOI:** 10.1186/s12885-017-3523-y

**Published:** 2017-08-14

**Authors:** Hao Huang, Xue-Ping Wang, Xiao-Hui Li, Hao Chen, Xin Zheng, Jian-Hua Lin, Ting Kang, Lin Zhang, Pei-Song Chen

**Affiliations:** 1 Department of Laboratory Medicine, The First Affiliated Hospital of Sun Yat-sen University, Guangzhou, Guangdong, 510060 People’s Republic of China; 2 Department of Laboratory Medicine, State Key Laboratory of Oncology in South China, Collaborative Innovation Center for Cancer Medicine, Sun Yat-sen University Cancer Center, Guangzhou, Guangdong 510060 People’s Republic of China; 3 Guangdong Esophageal Cancer Institute, Guangzhou, Guangdong People’s Republic of China

**Keywords:** Esophageal squamous cell carcinoma, Alanine aminotransferase/aspartate aminotransferase ratio, Gamma glutamyltransferase, Prognosis, Overall survival

## Abstract

**Background:**

The levels of liver function tests (LFTs) are often used to assess liver injury and non-liver disease-related mortality. In our study, the relationship between pretreatment serum LFTs and overall survival (OS) was evaluated in esophageal squamous cell carcinoma (ESCC) patients.

**Methods:**

Our purpose was to investigate the prognostic value of the preoperative alanine aminotransferase/aspartate aminotransferase (ALT/AST) ratio and gamma glutamyltransferase (GGT) in ESCC patients. A retrospective study was performed in 447 patients with ESCC, and follow-up period was at least 60 months until death. The prognostic significance of serum LFTs were determined by univariate and multivariate Cox hazard models.

**Results:**

LFTs including ALT, AST, LSR, GGT, TBA and LDH were analyzed. Serum LSR (HR: 0.592, 95% CI = 0.457–0.768, *p* < 0.001 and GGT (HR: 1.507, 95% CI = 1.163–1.953, *p* = 0.002) levels were indicated as significant predictors of OS. The 5-year OS among patients with higher LSR levels was longer compared with those patients with decreased LSR levels, not only in the whole cohort but also in the subgroups stratified by pathological stage (T1–T2 subgroup, T3–T4 subgroup, N0 subgroup and M0 subgroup). We also found that patients with a higher GGT might predict worse OS than patients with a normal GGT, not only in the whole cohort but also in the subgroups stratified by pathological stage (T3–T4 subgroup and N1-N2 subgroup).

**Conclusions:**

Both increased levels of LSR and decreased levels of GGT might predict shorter overall survival in ESCC patients. Our findings suggest that serum LSR and GGT levels could be used as a key predictor of survival in patients with ESCC.

## Background

Esophageal cancer is one of the most prevalent malignant diseases worldwide, among all histologic types, esophageal squamous cell carcinoma (ESCC) occupies major portion [1, 2], and has the sixth mortality rates of any cancer globally. Most patients with ESCC will receive optional therapeutic options, such as surgery only, surgery with adjuvant chemotherapy, and adjuvant systemic therapy which may consist of radiotherapy, or a combination of these treatments. However, the overall 5-year survival rate of patients treated with only surgical resection is less than 20% [3, 4]. As treatment plans are becoming more individualized for each patient, it is important to assess disease progression in a timely manner while accurately evaluating the prognosis [5]. To date, various serum biomarkers, such as SCC, CYFRA21-1and CEA, have been served as the valuable markers to estimate the prognosis of ESCC patients [6]. However, the sensitivity and specificity are not sufficient or reliable. Furthermore, correlations between ESCC survival and thrombin time (TT) [7] or apolipoprotein A1 (Apo-A1) [8] have been reported in our previous study. Thus, in order to improve the posttreatment survival of patients, identification of more effective and accurate biomarker of ESCC is a necessity.

The routine blood sample that examines liver function tests (LFTs), partly consist of alanine aminotransferase (ALT), aspartate aminotransferase (AST), the level of ALT/AST ratio (LSR), total bile acid (TBA), gamma glutamyltransferase (GGT), and lactate dehydrogenase (LDH). LFTs are often included as routine tests for many different liver and non-liver diseases and are often obtained at initial consultation. Furthermore, changes in LFTs levels in cancer patients before and after neoadjuvant treatment are closely related to postoperative recurrence, such as breast cancer [9], gastric adenocarcinoma and other cancers. Serum ALT and AST are the circulating transaminases in the body, and are specific markers of liver dysfunction, which can generate products in gluconeogenesis and amino acid metabolism through catalyzing the transfer of amino groups [10, 11]. Many studies have indicated that serum levels of ALT and AST may be correlated with hepatitis tumors [12], type 2 diabetes mellitus [13], cardiovascular disease [14] and other diseases, and is the level of ALT/AST ratio (LSR). However, no studies were used to evaluate the relationship between the pretreatment serum LSR and survival of ESCC patients. Furthermore, as a key enzyme in glutathione (GSH) metabolism, GGT is the major antioxidant of the cell, which have played a key role in neutralizing reactive oxygen compounds and free radicals by catalyzing the degradation of extracellular GSH [15]. Previous studies have reported on the associations of serum GGT levels with the risk of cancer [16, 17]. This study was designed to conduct a retrospective cohort analysis to explore the predictive role of the LSR and GGT on overall survival (OS) in patients with ESCC.

## Methods

### Patients

A total of 447 eligible patients (346 men and 101 women) confirmed as ESCC at the Sun Yat-sen University Cancer Center, China, were identified in the present study from January 2007 to July 2010. The main clinical characteristics are described in Table 1. The inclusion and exclusion criteria were included in our previous study especially for LFTs [8]. All patients were pathologically confirmed as ESCC, and exclusion criteria were as follows: (1) patients who received any drugs known to affect LFTs or surgery before being enrolled in this study; (2) patients who were diagnosed with liver diseases, cardiovascular disease, diabetes or metabolic syndrome, which could influence the serum LFTs; (3) patients with other types of tumors. In addition, the pathological stage of tumor was evaluated using the American Joint Committee on Cancer Staging system (AJCC, 2002; Greene) [18]. Only the first record of hospitalizations were retained, and all the patients had undergone treatment. Clinical data, such as demographic data, pathological stage (pTNM), alcohol consumption, therapeutic schemes, survival status and the levels of LFTs were available for all patients. The alcohol index was classified into ‘drinking’ and ‘not drinking’. All 447 patients underwent surgical resection. Specifically, 53.2% (238/447) patients underwent tumor resection only, and 3.8% (17/447) patients received unknown therapy. Furthermore, 36.2% (162/447) ESCC patients experienced chemotherapy after surgery, while 6 patients experienced radiation and 24 patients experienced both radiation and chemotherapy after surgery. Treatment strategies were determined by the pathological stage, the doctors’ opinions and the patient wishes. Primary esophagectomy and regional lymphadenectomy was included in this study as surgical procedure [19]. As adjuvant chemotherapy, a two-drug regimen of platinum-based drugs were administered for 4–6 cycles. Postoperative radiation of 46–64 Gy was mainly applied to the anastomosis, supraclavicular, and mediastinal lymphatics.

**Table 1:** Main clinical characteristics and parameters in 447 patients with ESCC

Characteristics	Median (25th–75th percentile) or no. (%)
Gender (n)	
Male	346 (77.4)
Female	101 (22.6)
Age	
< 59 years	233 (52.1)
≥ 59 years	214 (47.9)
Alcohol (n)	
No	261 (58.4)
Yes	186 (41.6)
Tumor location	
Upper	41 (9.2)
Middle	278 (62.2)
Lower	128 (28.6)
Stage (n)	
I and II	235 (52.6)
III and IV	212 (47.4)
Treatment (n)	
Surgery only	238 (53.2)
Surgery and chemotherapy	179 (40.0)
Surgery and radiotherapy	6 (1.3)
Surgery and chemotherapy and radiotherapy	24 (5.4)
Deads (n)	
No	199 (44.5)
Yes	248 (55.5)
Tests	
GGT	23.1 (16.8–35.2)
LDH	154.4 (135.7–174.2)
TBA	4.14 (2.43–6.65)
ALT	15.1 (11.5–20.8)
AST	18.2 (15.8–22.1)
ALT/AST	0.82 (0.67–1.02)

### Assessment of LFTs and follow-up

Patients received routine serum tests for biochemical detection at the first visit in our hospital. Serum samples were collected between 7 and 8 a.m., and were centrifuged at 3500 g/min for 8 min to allow serum separation. The levels of ALT, AST, GGT, TBA, and LDH were measured in serum using a Hitachi 7600 automatic biochemical analyzer (Hitachi High-Technologies, Tokyo, Japan). The LSR was calculated as the serum ALT level divided by the serum AST level.

Prior to use of these sera, all patients signed an informed consent. The follow-up method was same as that of our previous study [8]. The last follow-up session was in January 2016. This study was conducted with the approval of the Institute Research Ethics Committee of the Sun Yat-Sen University Cancer Center, Guangzhou, China.

### Statistical analysis

Data analyses were done using SPSS 16.0 for Windows software (IBM, Chicago, IL, USA). OS was calculated between the first diagnosis of ESCC and death, or final clinical follow-up. Data were expressed as the median. A Cox proportional-hazard model for multivariable analysis was applied for variables that proved to be significant in the univariate analysis. The Kaplan-Meier method and the log-rank test were used to plot the survival curves of this survey. The correlation between LSR, GGT and clinical characteristics was analyzed using the Mann–Whitney U test and χ^2^ test. A two tailed *P* value <0.05 was considered statistically significant.

## Results

### Patient characteristics

From January 2007 to July 2010, 447 patients had pathologically confirmed ESCC were recruited in this study. Pretreatment serum ALT, AST, GGT, TBA, and LDH levels were confirmed in all patients. Patient characteristics are summarized in Table 1. Median age was 59 years, and 77.4% of patients were males. The pathological stage of I-II and III-IV were observed in 235 (52.6%) and 212 (47.4%) patients, respectively. 238 (53.2%) of the 447 patients underwent surgery only (155 patients in stage I–II and 83 patients in stage III-IV), and 192 of these patients received the comprehensive treatment scheme (162 (36.2%) with surgery and chemotherapy; 6 (1.3%) with surgery and radiotherapy; 24 (5.4%) patients with surgery, chemotherapy, and radiotherapy), and 17 (3.8%) patients with unknown treatment after surgery. The follow-up period was from 1 month to 5 years, there were 199 patients still alive and 248 cancer-related deaths at the last clinical follow-up session.

### Univariate and multivariate analyses of prognostic factors

To estimate the predict value of the pretreatment LFTs in ESCC, the clinical datum (including age, gender, alcohol index, T classification, node metastasis, M status, TNM stage, histological differentiation, and treatment strategies) and the LFTs were included for univariate and multivariate analysis. In univariate analysis, significantly associations between clinical stage (HR: 3.132, 95% CI = 2.048–4.074, *p* < 0.001), treatment strategies (HR: 0.223, 95% CI = 1.050–1.425, *p* < 0.001), alcohol index (HR: 1.346, 95% CI =1.049–1.728, *p* = 0.020), GGT (HR: 1.395, 95% CI = 1.085–1.793, *p* = 0.01) and LSR (HR: 0.615, 95% CI = 0.478–0.792, *p* < 0.001) and overall survival were found, whereas age, gender, histological differentiation, LDH, and TBA were not significantly associated with OS (Table 2).

**Table 2:** Univariate and multivariate cox hazards analysis for overall survival in 447 patients with ESCC

	Univariate analysis		Multivariate analysis			
Variables	HR	95% CI	*p* value**	HR	95% CI	*p* value**
Gender						
Male vs. Female	1.119	0.837–1.497	0.448	0.876	0.679–1.131	0.310
Age (years)						
< 59 vs. ≥ 59	0.985	0.768–1.264	0.905	1.124	0.838–1.506	0.435
Histological differentiation (129)						
Differentiated vs. Undifferentiated	1.242	0.908–1.701	0.176			
T classification						
T3–4 vs. T1–2	1.851	1.356–2.525	0.000			
N classification						
Yes vs. No	3.029	2.324–3.948	0.000			
Metastasis						
Yes vs. No	4.049	2.308–7.103	0.000			
TNM stage						
III-IV vs. I-II	3.132	2.048–4.074	0.000	2.956	2.269–3.852	0.000
Tumor location						
Upper vs. Middle vs. Lower	1.215	0.984–1.501	0.071			
Alcohol history						
Yes vs. No	1.346	1.049–1.728	0.020	1.238	0.950–1.612	0.114
Treatment(n)						
Surgery only	1.235	1.063–1.434	0.006	1.083	0.953–1.231	0.221
Surgery and chemotherapy						
Surgery and radiotherapy						
Surgery and chemotherapy and radiotherapy						
Tests						
GGT						
≥ 23.1 vs. <23.1	1.395	1.085–1.793	0.01	1.507	1.163–1.953	0.002
LDH						
≥ 154.4 vs. <154.4	1.046	0.815–1.344	0.722			
TBA						
≥ 4.14 vs. < 4.14	1.172	0.912–1.506	0.215			
ALT						
≥ 15.1 vs. < 15.1	0.718	0.558–0.924	0.01	0.913	0.658–1.268	0.588
AST						
≥ 18.2 vs. <18.2	1.021	0.795–1.312	0.87			
ALT/AST(LSR)						
≥ 0.82 vs. <0.82	0.615	0.478–0.792	0.000	0.592	0.457–0.768	0.000

*HR* Hazard ratio, *95% CI* 95% confidence interval

**Cox hazard regression model

Multivariate analysis (Cox proportional hazards model) was carried out including all the variables identified as predictors of OS in the aforementioned univariate analysis, to evaluate whether these variables could be used as independent predictive factor for ESCC. The results revealed that pathological stage (HR: 2.956, 95% CI = 2.269 – 3.852, *p* < 0.001), GGT (HR: 1.507, 95% CI = 1.163 – 1.953, *p* = 0.002), and LSR (HR: 0.592, 95% CI = 0.457 – 0.768, *p* < 0.001) were independent and significant predictors for OS.

### Prognostic significance of LSR and GGT according to pathological stage and treatment strategies

**Fig. 1:**
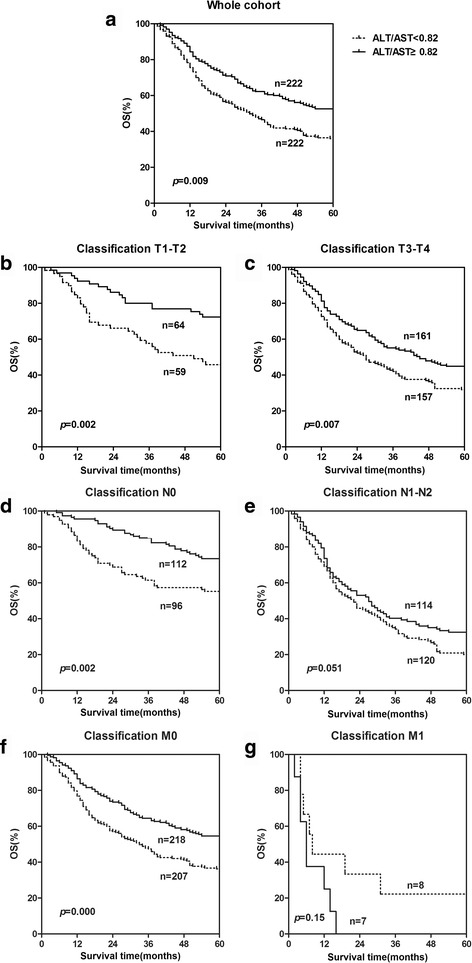
The prognostic value of serum LSR in ESCC in whole cohort and different pathological stages. Kaplan-Meier survival curves indicating lower LSR level was significantly related to poor survival not only in whole ESCC patients (**a**) but also in the subgroups stratified by pathological stage [T1–T2 subgroup (**b**), T3–T4 subgroup (**c**), N0 subgroup (**d**) and M0 subgroup (**f**)]. The OS was not significantly different in the N1–N2 subgroup (1**e**) and M1 subgroup (**g**)

**Fig. 2:**
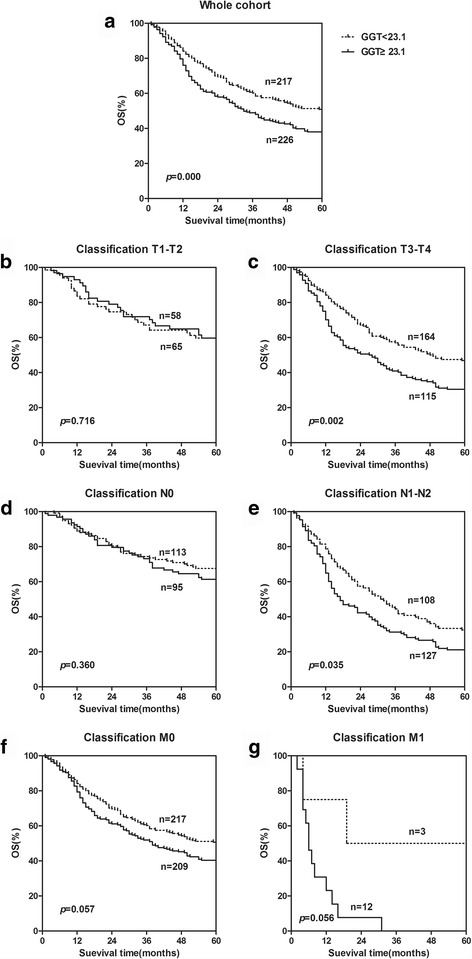
The prognostic value of serum GGT levels in ESCC in whole cohort and different pathological stages. Kaplan-Meier survival curves indicating higher GGT level was significantly related to shorter survival not only in whole ESCC patients (**a**) but also in the subgroups stratified by pathological stage [T3–T4 subgroup (**c**) and N1-N2 subgroup (**e**)]. ﻿The OS was not significantly different in the stage T1–T2 subgroup (**b**), N0 subgroup (**d**), M0 subgroup (**f**) and M1 subgroup (**g**)

In the Kaplan-Meier analysis, LSR and GGT type was closely associated with OS (Fig. 1a, Fig. 2a). In the whole cohort, the OS in patients with higher LSR (mean, 42.3 months) was 7.9 months longer compared with those with lower LSR type (mean, 34.4 months), and cumulative 5-year survival rate were 65.4% vs. 35.9% (higher LSR group, lower LSR group, respectively). Meanwhile, the OS was 5.4 months longer in patients with lower GGT (mean, 35.7 months) compared with those with higher GGT type (mean, 41.1 months), and the cumulative 5-year survival rate were 50.45% vs. 38.7% (lower GGT group, higher GGT group, respectively).

As an important predictor factor for OS, the relationship between LSR, GGT and survival was further evaluated according to the pathological stage. Patients with decreased LSR levels (<0.82) presented a significantly poor OS compared with those patients with increased LSR levels both in the T1–T2 subgroup (*p* = 0.002, Fig. 1b), T3–T4 subgroup (*p =* 0.007, Fig. 1c), N0 subgroup (*p =* 0.002, Fig. 1d), and M0 subgroup (*p* < 0.001, Fig. 1f). The OS was not significantly different in the N1–N2 subgroup (*p =* 0.051) (Fig. 1e) and M1 subgroup (*p* < 0.15) (Fig. 1g). Patients with decreased GGT levels (<23.1) presented a significantly poor OS compared with those patients with increased GGT levels only in the stage T3–T4 subgroup (*p* = 0.002, Fig. 2c) and N1-N2 subgroup (*p* = 0.035, Fig. 2e). The OS was not significantly different in the stage T1–T2 subgroup (*p* = 0.716) (Fig. 2b), N0 subgroup (*p =* 0.360) (Fig. 2d), M0 subgroup (*p* = 0.057) (Fig. 2f) and M1 subgroup (*p* = 0.056) (Fig. 2g).

### The relationship between LSR, GGT concentrations and clinicopathologic factors in ESCC patients

Further analysis of the associations between serum LSR, GGT concentrations and clinicopathologic variables in ESCC patients are shown in Tables 3 and 4. LSR was associated with alcohol consumption (*p =* 0.024), tumor location (*p =* 0.038) and death (*p* < 0.001). In contrast, there were no significant associations between LSR and other clinicopathologic variables, including gender (*p =* 0.165), age (*p =* 0.092), pathological stage (*p =* 0.282) and treatment scheme (*p =* 0.109). GGT concentration was significantly associated with gender (*p =* 0.001), alcohol consumption (*p* < 0.001), tumor location (*p =* 0.002), pathological stage (*p =* 0.007), treatment scheme (*p =* 0.033) and death (*p =* 0.034), although age was not significant (*p =* 0.056).

**Table 3:** Relationship between the LSR levels and clinical characteristics of patients with ESCC

Variables	LSR					
	Cases (n)	≥0.82	<0.82	*p* value*	Mean ± SD	*p* value**
Gender (n)						
Male	342	179	163	0.305	0.89 ± 0.34	0.165
Female	101	47	54		0.84 ± 0.27	
Age						
< 59 years	232	122	110	0.448	0.91 ± 0.35	0.092
≥ 59 years	211	104	107		0.85 ± 0.27	
Alcohol						
No	259	122	137	0.051	0.85 ± 0.31	0.024
Yes	184	104	80		0.92 ± 0.34	
Tumor location						
Upper	40	25	15	0.011	0.99 ± 0.39	0.038
Middle	275	131	175		0.86 ± 0.33	
Lower	128	70	58		0.88 ± 0.28	
Stage						
I and II	234	125	109	0.284	0.89 ± 0.32	0.282
III and IV	209	101	108		0.87 ± 0.33	
Treatment						
Surgery only	236	117	119	0.397	0.85 ± 0.31	0.109
Surgery and chemotherapy	177	97	80		0.92 ± 0.34	
Surgery and radiotherapy	6	3	3		0.89 ± 0.25	
Surgery and chemotherapy and radiotherapy	24	9	15		0.81 ± 0.34	
Deaths						
No	198	120	78	0.000	0.96 ± 0.35	0.000
Yes	245	106	139		0.82 ± 0.29	

Mean ± SD, Mean ± standard deviation

*P values were calculated using the chi-squared test (χ^2^ test), *p* < 0.05 indicated significant differences

**P values were calculated using unpaired Student’s t-tests or Mann–Whitney U test, *p* < 0.05 indicated significant differences

**Table 4:** Relationship between the GGT concentration and clinical characteristics of patients with ESCC

Variables	GGT					
	Cases (n)	≥23.1	<23.1	*p* value*	Mean ± SD	*p* value**
Gender						
Male	343	184	158	0.004	37.96 ± 4.72	0.001
Female	101	38	63		26.20 ± 1.84	
Age						
< 59 years	232	124	108	0.128	39.85 ± 5.20	0.056
≥ 59 years	212	98	114		30.29 ± 2.85	
Alcohol						
No	260	97	163	0.000	27.57 ± 3.06	0.000
Yes	184	125	59		46.18 ± 5.37	
Tumor location						
Upper	40	21	19	0.009	40.24 ± 5.29	0.002
Middle	276	123	153		30.05 ± 2.60	
Lower	128	78	50		45.02 ± 6.22	
Stage						
I and II	234	106	128	0.037	29.81 ± 3.43	0.007
III and IV	210	116	94		41.39 ± 4.98	
Treatment (n)						
Surgery only	236	102	134	0.013	31.64 ± 3.84	0.033
Surgery and chemotherapy	178	106	72		39.09 ± 4.72	
Surgery and radiotherapy	6	3	3		33.73 ± 3.20	
Surgery and chemotherapy and radiotherapy	24	12	12		43.35 ± 4.81	
Deaths						
No	198	86	112	0.013	31.82 ± 3.83	0.034
Yes	246	136	110		38.07 ± 4.58	

Mean ± SD, Mean ± standard deviation

*P values were calculated using the chi-squared test (χ^2^ test), *p* < 0.05 indicated significant differences

**P values were calculated using unpaired Student’s t-tests or Mann–Whitney U test, *p* < 0.05 indicated significant differences

## Discussion

In ESCC, prognostic biomarkers are needed to understand the development of cancer and for tailoring individual therapeutic strategies. Thus, it is imperative that inexpensive and convenient prognostic biomarkers for this disease are identified.

LFTs are routine laboratory tests, and earlier studies have noted the relationship of LSR, GGT and the risk of malignances, such as breast cancer [9], gastric cancer [20], liver cancer and other cancers. To date, the associations between LSR, GGT and ESCC survival have not been well developed. In our study, the cut-off points of LSR and GGT were defined as the median, which demonstrated that pretreatment serum LSR and GGT levels were related with 5-year OS in ESCC patients. Among the 447 ESCC cases examined in this retrospective study from Sun Yat-Sen University Cancer Center, we observed that patients with higher LSR levels showed significantly better prognosis compared with those patients with low LSR levels, not only in the entire cohort but also in the subgroups stratified by pathological stage (T1–T2 subgroup, T3–T4 subgroup, N0 subgroup and M0 subgroup). We also found that patients with a higher GGT showed significantly poorer prognosis than normal GGT patients, not only in the entire cohort but also in the subgroups classified by pathological stage (T3–T4 subgroup and N1-N2 subgroup). After adjustment for clinical characteristics, the elevated serum LSR and GGT were both associated with alcohol index and death.

The LSR and GGT tests are simple, inexpensive and widely used in clinical laboratories. As the major critical enzymes, ALT and AST generate products in gluconeogenesis and amino acid metabolism, and as specific markers of liver dysfunction, they catalyse the transfer of amino groups [10]. GGT is the major enzyme in the glutathione (GSH) catabolism, which also function as a biomarker for excessive alcohol intake [15]. Furthermore, many studies have demonstrated that LFTs have effects on the different hepatic injures, type 2 diabetes mellitus, cardiovascular disease and other diseases. In addition, several reports have confirmed the links between LFTs and prognosis in cancer patients. Chen el al. reported that the lower preoperative LSR level was found to be associated with decreased survival in patients with gastric adenocarcinoma [20]. Shen also suggested that pretreatment AST was related with the clinical outcome in hepatitis B-induced hepatocellular carcinoma after hepatectomy [21]. Preyer et al. identified that GGT was an independent risk factor in breast cancer over and above the alcohol consumption and other life style risk factors [16]. Mok et al. published a large study of new cancer cases, which occurred among 1,662,087 Koreans (ages 20–95 years, 1108,121 male and 553,966 female) who received health insurance from the National Health Insurance Service during 1995 and 1998. These patients were followed up for 17 years, and the elevated serum level of GGT was independently links with risk of various tumors, such as colorectal, stomach, lung and bile duct cancer [22]. However, the function of LSR and GGT in carcinogenesis is not well understood. It is interesting to consider the reason about the observed links between LSR, GGT levels and incident cancer risk, which may be attributed to its multiple properties, including anti-inflammatory and antioxidant properties. Moreover, serum LFTs are markers of alcohol intake, especially GGT. In our retrospective study, higher LSR and GGT levels were related to ESCC patients consumption of alcohol, and a significant relationship between increased GGT and alcohol intake has previously been reported [23, 24]. Numerous studies have indicated that alcohol consumption have influence on the risk of cancers of the oral cavity and pharynx, esophagus, stomach, liver and colorectum [25]. The possible mechanisms suggested include acetaldehyde being carcinogenic, mutagenic, influencing the ability to bind to DNA and protein, and destroying folate which results in secondary hyper-regeneration [26].

Cancer is a pro-inflammatory state, in which inflammatory cells actively participate in the occurrence of tumor development, such as tumor cell proliferation, survival, and migration [27–29]. For LSR, the exact mechanisms underlying the association remain unclear: one explanation is that the change in LSR levels is associated with subclinical inflammation, which may result in continued damage of tissue precipitating some noninfectious diseases [30–32]. The possible inference from LSR levels may be that they influence some of the pro-inflammatory mediators involved in carcinogenesis and affect tumor invasion and metastasis. Tumorigenic factors (inflammation and oxidative stress), which participate in these stages, have influenced on the cancer initiation in cascade of steps. The relationship between inflammation and cancer is very complex. Inflammatory factors were produced during the inflammatory response, including chemokines (e.g. CCL2, CXCL8) and cytokines (e.g. IL-6), which can recruit more inflammatory cells to the lesion site, such as neutrophilic granulocytes and mononuclear macrophages, and resulting in the inflammatory microenvironment [33, 34]. Moreover, as potent inflammatory mediators, TNF-α and IL-6 were released from stimulated T-cells and activated macrophages, which may result in continued damage to the liver tissue thereby influencing the LSR levels. [35]. The other possible explanation is that LSR levels may influence some of the pro-inflammatory mediators involved in carcinogenesis. Furthermore, through stimulating the growth of tumor cells and driving signaling transduction pathways, reactive oxygen factors leads to activation of redox-sensitive transcription species and genes which participated in the growth, proliferation, and survival of tumor cells.

Several potential mechanisms have been postulated for the relationship between GGT and cancer: 1. As the essential parts of the cellular defense apparatus, GGT and GSH have been used to against oxidative stress [15]. The elevated GGT levels in tumor cells can produce the reactive oxidant species (ROS), which may drive tumor progression; 2. Increased GGT has been regarded as a marker of exposure to certain carcinogens, which include persistent environmental pollutants including dioxins, lead, organochlorine pesticides and so on [36, 37]; 3. As a GGT isoenzyme, GGTII has been reported to a useful tumor marker in the diagnosis of small hepatocellular carcinoma [38]; 4. GGT levels can be affected by environmental and lifestyle factors (such as diet, smoking, and drinking) and genetic regulation [39]. However, further studied are needed to determine the underlying mechanisms.

Recently, serum markers have used in noninvasive cancer diagnosis, individual treatment, monitoring of prognosis and recurrence, and includes circulating tumor cells, exosomes, free DNA, and protein-based markers. As protein-based markers, the association between the LFTs and survival in ESCC were evaluated, and LSR and GGT were demonstrated to be independent predictors of overall survival in ESCC. Thus, LSR appears to be a new prognostic marker in ESCC.

## Conclusions

In summary, this retrospective study is the first to validating the association between pretherapy serum LFTs and ESCC. Based on the 447 patients in our cohort, we showed that pretreatment serum LSR and GGT levels are prognostic factors for OS in ESCC, both in the entire cohort and in subgroups classified by pathological stage. These biomarkers offer high reproducibility and could be tested easily in clinical laboratories. As our study is a retrospective analysis, it is only valid for generating the hypothesis, a large-scale and prospective study should be done to validated the value of LSR and GGT in HCC.

## Abbreviations

ALT: Alanine Aminotransferase
AST: Aspartate Aminotransferase
CEA: Carcino-embryonic antigen
CYFRA21-1: Cytokeratin 19 fragments
ESCC: Esophageal squamous cell carcinoma
GGT: Gamma glutamyltransferase
GSH: Glutathione
LDH: Lactate dehydrogenase.
LFTs: liver function tests
LSR: ALT/AST Ratio
SCC: Squamous cell carcinoma antigen
TBA: Total bile acid
